# Complementary or alternative? The use of homeopathic products and antibiotics amongst pre-school children

**DOI:** 10.1186/1471-2296-9-8

**Published:** 2008-01-30

**Authors:** Lesley Wye, Alastair D Hay, Kate Northstone, Jackie Bishop, Judith Headley, Elizabeth Thompson

**Affiliations:** 1 Academic Unit of Primary Health Care, Community Based Medicine, University of Bristol, Cotham House, Cotham Hill, Bristol, UK; 2 ALSPAC, Social Medicine, University of Bristol, Tyndall Ave, Bristol, UK; 3 Bristol Homeopathic Hospital, Bristol Royal Infirmary, Cotham Hill, Bristol, UK

## Abstract

**Background:**

Any intervention to reduce the inappropriate use of antibiotics for infections in children has the potential to reduce the selective pressure on antimicrobial resistance and minimise the medicalisation of self-limiting illness. Little is known about whether homeopathic products might be used by some families as an alternative to antibiotics or the characteristics of such families. We used the Avon Longitudinal Study of Parents and Children (ALSPAC) observational dataset to explore the hypothesis that the use of homeopathic products is associated with reduced antibiotic use in pre-school children and to identify characteristics of the families of pre-school children given homeopathic products.

**Methods:**

Questionnaires data were completed by the parents of 9723 children while aged between 3–4.5 years in Bristol UK. Univariable and multivariable analyses were used to explore the relationships between antibiotic and homeopathic product use.

**Results:**

Six percent of children had received one or more homeopathic products and 62% one or more antibiotics between the ages of 3 and 4.5 years. After adjustment for factors associated with antibiotic use, there was no association between homeopathic product and antibiotic use (adjusted OR = 1.02, 95% CI 0.84, 1.24). Factors independently associated with child homeopathic product use were: higher maternal education, maternal use of homeopathic products, maternal lack of confidence in doctors, mothers reporting that they were less likely to see doctor when the child was ill, children being given vitamins, watching less television and suffering from wheeze and food allergies.

**Conclusion:**

In this observational study, the use of homeopathic products was not associated with decreased antibiotic consumption, suggesting the use of homeopathic product complements rather than competes with the use of antibiotics in pre-school children. The characteristics of mothers giving homeopathic products to their children are similar to those associated with adult self-administration.

## Background

### Medicine, antibiotic and health service use amongst pre-school children

The use of medicines in children has now risen to the top of the political and research agendas [1,2]. Regarding antibiotics in particular, pre-school children consume more than any other age group [3]. In 1996, a study found that up to 57% and 54% of pre school boys and girls respectively had received an antibiotic [4] and studies since have shown that UK prescribing is still up to 30% higher than our Northern European counterparts [5].

Inappropriate or unnecessary use of antibiotics for viral infections in children causes concern for policy makers, commissioners, clinicians, complementary therapists and parents [6-8] for two reasons. First, because it may promote the transmission of both antibiotic susceptible and resistant bacteria and infections in the community [9,10]. And second, because it incorrectly reinforces the need for parents to consult for similar illnesses in the future, a process known as illness "medicalisation" [11]. Furthermore, children under five are amongst the greatest users of NHS services [12], principally for respiratory tract infections [13]. While important, strategies to maximize the appropriate use of NHS services also need to ensure that if alternative complementary therapies such as homeopathy are available and used by parents, that serious conditions of childhood are not missed. Thus infectious disease in pre-school children and the use of antibiotics have important public health and resource implications.

### Complementary and alternative use

The growing literature on complementary and alternative medicine (CAM) puts estimates of adult use between 10–50% depending on the definition of CAM applied [14-16]. CAM users are more likely to be better-educated women aged between 30–55, who are economically secure and less satisfied with conventional medicine [15,17]. Far less is known about children's use of CAM [18]. Estimates of use in hospital population ranges from 1.8%–51% [19-22], with two studies finding that children in hospital use CAM as an adjunct to, rather than a replacement of, conventional medicine [20,22]. A community based study in England of 1230 children found that 17.9% of children had used some form of CAM, mostly for ear, nose and throat or dermatological conditions and nearly half of these children had one or both parents who also used CAM [23].

In looking at children's use of homeopathy, a range of types of use exists from self-prescribed over the counter products to practitioner prescribed by a professional or medically trained homeopath. A combined Welsh/Australian hospital survey found that 8% of the Cardiff children and 10.5% of the Melbourne population used homeopathic products, but the source of prescription was unclear [21]. Within a primary care population, a study of medical homeopaths from the Bristol Homeopathic Hospital found that almost 20% of their patients over a six year period were under the age of 16 [24]. A Scottish study found that 49% of practices in Scotland prescribed homeopathy in 2003–2004 with the highest prevalence amongst children under 1 year of age (9.5/1000) [25]. Another Scottish study found GPs prescribed homeopathy most commonly for colic, cuts and bruises, teething, dermatological conditions, earache, influenza and upper respiratory tract infections [26].

Some qualitative studies suggest that parents may be using homeopathy instead of conventional medicine for their children. An ethnographic study of committed adult users in South London found that they consulted their GP for diagnosis but took homeopathic products for treatment because antibiotics and other drugs were seen as weakening the immune system and counteracting the remedy [27]. Many of these participants were mothers who made health decisions for their children using this same set of beliefs. A recent qualitative study in Norway also found the same [28].

### Study aims

Given the need to promote the judicious use of antibiotics and the lack of knowledge about children's homeopathic product consumption, the aims of this study were to use observational data to investigate if homeopathic product users consumed fewer antibiotics and to describe the characteristics of pre-school children given homeopathic products.

## Methods

The Avon Longitudinal Study of Parents and Children (ALSPAC) is an ongoing prospective, population-based study, which recruited pregnant women resident in the former Avon Health Authority area, who had an estimated delivery date between 1st April 1991 and 31st December 1992. The majority of the known births from the geographically defined catchment area were included, resulting in a total cohort of 13971 children surviving to one year of age. This study population has social and demographic characteristics in common with national census surveys. More detailed information can be found on the ALSPAC website [29].

The main source of data collection in ALSPAC is through questionnaires completed by primary carers at various time points during pregnancy and throughout their child's life. Data on the use of antibiotic and homeopathic products by children and their parents have been collected since its inception. Ethical approval for the study was obtained from the ALSPAC law and ethics committee and the three local research ethics committees.

Mothers recorded their own use of homeopathic products in an antenatal questionnaire at 18 weeks. At 4.5 years (54 months), primary carers completed a questionnaire that asked about the child's use of various medications during the preceding 18 months. They recorded their child's use of antibiotics and homeopathic products as well as cough medicines, eye and ear drops and skin creams. They were asked to indicate whether they had been used just for 'one episode' or for 'two or more episodes' (tick box) and where possible to record the names of the products used (text box). Both tick and text box data were used to determine antibiotic and homeopathic product use. Antibiotic and homeopathic users were defined as those who ticked 'yes' to antibiotic or homeopathic product use respectively and/or provided relevant text data in other parts of the questionnaire.

All responses were checked for accurate completion of the antibiotic fields using coding procedures reported elsewhere [30]. For the homeopathic data, two homeopaths and a herbalist classified text where it was not immediately clear if homeopathic or herbal products had been used. Responses were excluded if a positive response to the homeopathic question was given but text data indicted that this was erroneous (n = 20). Data were not available on the conditions for which the antibiotics and homeopathic products were used, but the dataset was refined to exclude creams, ointments, Bach flower remedies and Arnica as they are not used to treat infections.

To learn more about the characteristics of children who use homeopathic products, a variety of possible factors of homeopathic product use, which were also potential confounders in the relationship between antibiotic and homeopathic product use, were identified by the study team and used in the multivariable logistic regression analysis (see Tables 1 and 2).

**Table 1:** Association between homeopathic and antibiotic use at 54 months

	**Antibiotic use**			
**Homeopathic use**	No (38.3%)	Yes (61.7%)	Crude OR	Adjusted OR

No (94%)	3525 (38.6%)	5619 (61.4%)	1.23 (1.03, 1.46)	1.02 (0.84, 1.24)
Yes (6%)	196 (33.9%)	383 (66.1%)		

Adjusted for factors found to be associated with antibiotic use (p < 0.05) namely child's general health, Mum contacts GP when child ill, maternal anxiety, child's use of vitamins, child had wheezing, child had earache, child had cough

**Table 2:** Factors associated with homeopathic product use in pre-school children

		Unadjusted homeopathic product use			Adjusted OR
Factor (survey time-point)	Categories	Yes	No	P value	

Maternal education (54 months)	CSE or less	29 (1.9%)	1495 (98.1%)	p < 0.0001	1.0
	Vocational	18 (2.1%)	842 (97.9%)		1.04 (0.53, 2.03)
	School O level	133 (4%)	3223 (96.0%)		1.65 (1.03, 2.64)
	A level	222 (9.7%)	2070 (90.3%)		3.63 (2.28, 5.78)
	Degree	158 (11.6%)	1209 (88.4%)		3.56 (2.20, 5.76)
Maternal age (54 months)	< 19	5 (1.9%)	263 (98.1%)	p < 0.0001	
	20–24	48 (3.2%)	1449 (96.8%)		
	25–29	188 (4.9%)	3688 (95.1%)		
	30–34	220 (7.3%)	2803 (92.7%)		
	35+	118 (11.1%)	943 (88.9%)		
Housing tenure (54 months)	Owner/occupied	455 (6.4%)	6612 (93.6%)	p < 0.0001	1.0
	Council/Housing	29 (2.7%)	1062 (97.3%)		0.94 (0.61, 1.45)
	Assoc	53 (9.9%)	485 (90.1%)		1.71 (1.21, 2.40)
	other				
Family income per wk (54 months)	<£ 100	26 (4.4%)	560 (95.6%)	p < 0.0001	*
	£ 100–£ 199	55 (4.6%)	1151 (95.4%)		
	£ 200–£ 299	103 (4.9%)	2004 (95.1%)		
	£ 300–£ 399	115 (6.4%)	1690 (93.6%)		
	£ 400+	192 (8.4%)	2105 (91.6%)		
Maternal smoking cigarettes per day (47 months)	None	427 (6.3%)	6327 (93.7%)	p < 0.0001	*
	1–9	52 (9.7%)	482 (90.3%)		
	10+	50 (3.7%)	1311 (96.3%)		
Maternal anxiety (47 months)	Yes	156 (7.4%)	1956 (92.6%)	p = 0.004	*
	No	384 (5.7%)	6366 (94.3%)		
Child's fruit/veg consumption score (54 months)	1	22 (4.5%)	472 (95.5%)	p = 0.006	*
	2	69 (4.5%)	1450 (95.5%)		
	3	188 (5.9%)	3011 (94.1%)		
	4	284 (6.8%)	3905 (93.2%)		
Hrs spent watching tv daily (54 months)	<1	214 (8.9%)	2178 (91.1%)	p < 0.0001	*
	1–2	230 (5.9%)	3688 (94.1%)		
	2+	115 (3.7%)	2958 (96.3%)		
Child had vitamins since 3 years of age (54 months)	Yes	364 (10.5%)	3119 (89.5%)	p < 0.0001	2.55 (2.09, 3.11)
	No	215 (3.5%)	5993 (96.5%)		
Child's general health (42 months)	Very healthy	246 (4.7%)	8143 (95.3%)	p < 0.0001	*
	Sometimes ill/never well	290 (7.8%)	272 (92.2%)		
Food allergies (42 months)	Yes	83 (12%)	607 (88.0%)	p < 0.0001	2.07 (1.55, 2.76)
	No	496 (5.5%)	8539 (94.5%)		
Wheeze (42 months)	Yes	111 (8%)	1283 (92.0%)	p = 0.001	1.32 (1.03, 1.71)
	No	430 (5.6%)	7212 (94.4%)		
Earache (42 months)	Yes	198 (7%)	2620 (93.0%)	p = 0.005	*
	No	343 (5.5%)	5875 (94.5%)		
High temperature (42 months)	Yes	364 (6.4%)	5349 (93.6%)	p = 0.044	*
	No	177 (5.3%)	3146 (94.7%)		
Dry itchy rash in joints (42 months)	Yes	154 (7.5%)	1893 (92.5%)	p = 0.001	*
	No	382 (5.5%)	6544 (94.5%)		
Mum has no confidence in doctors (54 months)	Feels exactly/often feels	57 (8.6%)	606 (91.4%)	p < 0.0001	1.54 (1.26, 1.89)
	Sometimes feels	233 (7.8%)	2740 (92.2%)		1.81 (1.30, 2.45)
	Never feels	237 (4.7%)	4781 (95.3%)		1.00
Mum contacts GP when child ill (54 months)	Always	48 (3.1%)	1503 (96.9%)	p < 0.0001	1.00
	Usually	155 (5.3%)	2785 (94.7%)		1.41 (0.98, 2.04)
	Sometimes	320 (7.7%)	3833 (92.3%)		1.87 (1.32, 2.65)
	Never	12 (8.1%)	136 (91.9%)		1.64 (0.73, 3.69)
Maternal use of homeopathic products (18 weeks antenatal)	Yes	82 (16.0%)	430 (84.0%)	p < 0.0001	4.72 (3.45, 6.44)
	No	217 (2.4%)	8996 (97.6%)		

* = factor not included in final model as p > 0.05

To determine the relationship between antibiotic and homeopathic product use and to identify the maternal and child characteristics of homeopathic product use, cross-tabulations were performed and the chi-square test was used to determine the strength of association of any observed differences. Logistic regression was performed to assess the independent effect of homeopathic product use on antibiotic use adjusting for potential confounders, namely factors found to be associated (p ≤ 0.05) with antibiotic and homeopathic product use in the chi-square analysis. Factors with a p ≤ 0.05 were retained in the models and adjusted odds ratios and 95% confidence intervals obtained. Multivariable logistic regression was also performed to identify those factors independently associated with homeopathic product use. Collinearity amongst the final set of explanatory variables was assessed by evaluating variance inflation factors. There was no evidence of collinearity based on these values.

## Results

Of the 13971 children originally recruited into the cohort, 12064 still remained in the study at the 4.5 year timepoint. From these, 9723 questionnaires were returned with data of interest complete giving a response rate of 80.3%. Of these, 61.7% (n = 5619) had used antibiotics between the ages of 3–4.5 years in 1995–1997. Six percent (n = 579) received a homeopathic product. On univariable analysis, children using homeopathic products were more likely to use antibiotics (OR 1.23, 95% CI: 1.03, 1.46). After controlling for other factors associated with antibiotic use, this association disappeared (OR 1.02, 95% CI: 0.84, 1.24) (see Table 1).

In the multivariable analysis, we found that children who used antibiotics were more likely to be reported to take vitamins and be perceived as suffering from poorer health, wheeze, earache and cough (all p < 0.0001). Mothers self-reported they were more likely to suffer from anxiety (p < 0.0001) and were more likely to contact the GP when their child was ill (p < 0.0001) (see Table 3).

**Table 3:** Factors associated with antibiotic use in pre-school children

		Unadjusted Antibiotic use			Adjusted OR
Factor (survey timepoint)	Categories	Yes	No	P value	

Homeopathic use (54 months)	Yes	383 (66.1%)	196 (33.9%)	p = 0.024	0.84 (0.65, 1.09)
	No	5619 (61.4%)	3525 (38.6%)		
Mother has use of car (33 months)	Yes, own	4924 (61.9%)	3025 (38.1%)	p = 0.039	*
	Yes, can borrow	120 (62.8%)	71 (37.2%)		
	No	305 (56.6%)	235 (43.5%)		
Mum contacts GP when child ill (54 months)	Always	1026 (65.2%)	525 (33.8%)	p < 0.0001	1.00
	Usually	1962 (66.7%)	978 (33.3%)		0.97 (0.85, 1.12)
	Sometimes	2375 (57.2%)	1778 (42.8%)		0.69 (0.60, 0.78)
	Never	57 (38.5%)	91 (61.5%)		0.35 (0.24, 0.50)
Mum asks chemist when child ill (54 months)	Always	15 (62.5%)	9 (37.5%)	p = 0.036	*
	Usually	160 (59.0%)	111 (41.0%)		
	Sometimes	3427 (62.8%)	2028 (37.2%)		
	Never	1818 (59.8%)	1224 (40.2%)		
What mum does with prescribed medicines (54 months)	Use all up	4346 (66.5%)	2194 (33.5%)	p < 0.0001	*
	Use until better	597 (51.0%)	573 (49%)		
	Save some	63 (54.8%)	52 (45.2%)		
	Share it	10 (62.5%)	6 (37.5%)		
	Back to doctor	36 (70.6%)	15 (29.4%)		
	stopped	28 (57.1%)	21 (42.9%)		
Maternal anxiety (47 months)	Yes	1380 (65.3%)	732 (34.7%)	p < 0.0001	1.15 (1.03, 1.29)
	No	4078 (60.4%)	2672 (39.6%)		
Child's general health (42 months)	Very healthy/mostly well	5289 (61.1%)	3368 (38.9%)	p < 0.0001	1.00
	Sometimes ill/never well	234 (79.6%)	60 (20.0%)		1.18 (0.85, 1.65)
Child had vitamins since age 3 (54 months)	Yes	2365 (67.9%)	1118 (32.1%)	p < 0.0001	1.46 (1.33, 1.61)
	No	3635 (58.6%)	2573 (41.4%)		
Child had wheeze (42 months)	Yes	1018 (73.0%)	376 (27.0%)	p < 0.0001	1.65 (1.44, 1.89)
	No	4558 (59.6%)	3084 (40.4%)		
Child had earache (42 months)	Yes	2168 (76.9%)	650 (23.1%)	p < 0.0001	2.70 (2.41, 3.02)
	No	3408 (54.8%)	2810 (45.2%)		
Child had cough (42 months)	Yes	4988 (62.9%)	2944 (37.1%)	p < 0.0001	1.11 (0.82, 1.49)
	No	588 (53.3%)	516 (46.7%)		
Child had high temperature (42 months)	Yes	3812 (66.7%)	1901 (33.3%)	p < 0.0001	*
	No	1764 (53.1%)	1559 (46.9%)		
Child had food allergies (42 months)	Yes	5522(61.1%)	3513(38.9%)	p < 0.0001	*
	No	480 (69.6%)	210 (30.4%)		
Child had dry itchy rash (42 months)	Yes	1317 (64.3%)	730 (35.7%)	p = 0.006	*
	No	4222 (61%)	2704 (39%)		

* = factor not included in final model as p > 0.05

Table 2 shows the results of multivariable analysis identifying the characteristics of homeopathic product use. Mothers who lived in rented properties and those with O levels or higher were more likely to have children who use homeopathic products (both p < 0.0001) as were mothers who sometimes or often had no confidence in their doctors or tended not to contact their GP when their child was ill (both p < 0.0001). Mothers who had used homeopathic products in pregnancy were four times more likely to give them to their child (p < 0.0001).

Children who had taken vitamins were more than twice as likely to take homeopathic products as were children suffering from food allergies. Children with wheeze were also more likely to be given homeopathic products (all p < 0.0001).

## Discussion

Sixty-two percent of the children in this study received an antibiotic between 1995 and 1997, which is slightly higher than other studies at this time [4]. We did not find evidence that homeopathic product use was associated with reduced antibiotic use. Six percent of the children in this population were given homeopathic products, which is compatible with the range found in other studies of children's CAM use.

### Study strengths and limitations

The strength of this study is that it is the only community based study that addresses the question of antibiotic and homeopathic product use in pre-school children. Nonetheless, these data are now over ten years old and it is possible that the situation has changed. However, it is difficult to determine the direction of change. We do know that antibiotic usage has decreased [31]. But there are no recent surveys of homeopathic product or complementary therapy use amongst children in the community in the UK, although a recent hospital based study in Wales found that 8% of the children in its sample had used homeopathic products, which is comparable to our results of 6% for prevalence [21]. Furthermore, even in surveys that do exist there are different definitions of homeopathy and complementary therapies, making it difficult to compare trends over time. Potentially, studies on expenditure offer further insight on prevalence of use, but they are also scarce. Interestingly, the only survey on expenditure that has been repeated robustly at three time points (1993, 2000, 2004) found that spending on complementary therapies in Australia actually decreased from 2000 to 2004 [32]. Thus, although the data for our study were collected over a decade ago and could be out of date, given the paucity of information on homeopathic product use amongst community populations, it makes an important contribution.

Like all studies using retrospective self-completion questionnaires, this study is subject to recall bias. This could have led to reporting of only the most severe or recent illness episodes and their associated treatment, in which case it may under-estimate antibiotic and homeopathic product use. Parents may also have under-reported what they felt was more acceptable e.g. fewer hours of television watching. Unfortunately, ALSPAC has not carried out any studies investigating the 'true' extent or direction of recall bias, so it is difficult to comment further. Response bias may also be present, leading to an over-estimate of antibiotic and homeopathic product use (as it is known that better educated, older mothers living in their own properties are more likely to complete questionnaires [33]).

Although details on type of homeopathic products were available, the limitations of the data meant we could not determine:

1. if the homeopathic products were over the counter or practitioner prescribed

2. if the episode of illness treated with a homeopathic product was the same as the episode in which an antibiotic was used

3. the conditions for which the children took homeopathic products or antibiotics

4. if the homeopathic products were used specifically for infections

### Implications of this study

The primary finding of this study, which has not been previously explored, is that parents offering their children homeopathic products were no less (or more) likely to use antibiotics than non-homeopathic product users, suggesting that the use of homeopathic products complements rather than competes with the use of antibiotics in pre-school children. Had we found evidence to support our original hypothesis, the implications for the reduction of population level antibiotic use could have been important, possibly suggesting that further, experimental, research into the promotion of homeopathic medicines as an alternative was warranted. From our data, we cannot determine the source of homeopathic products (local commercial outlets, NHS doctor homeopaths or professional practitioners). However a sister project looking at the same cohort of children just over two years later analysed information on source of prescription and found that nearly half of the children using homeopathic products obtained them from over the counter [34]. The impact of over the counter and practitioner prescribed homeopathic product use on antibiotic use could be the subject of future research.

In looking at the characteristics of those using homeopathic products, children with wheeze and food allergies were more likely to use homeopathy. Comparing the profile of general adult CAM users in the literature [14,35,36] to that of mothers who used homeopathic products for their pre-school children in this study, both groups share similar features, which is unsurprising as adults are making decisions in both cases. However, reported health seeking behaviours differed between mothers who gave their children antibiotics and mothers who gave their children homeopathic products. The mothers of children receiving antibiotics portray themselves as more likely to seek out NHS services. In contrast, the mothers of children who used homeopathic products reported they were less likely to turn to the NHS when their child was ill and had less confidence in doctors. Instead, they attempt to manage their child's health through self-help measures, of which homeopathic product use is one.

## Conclusion

The aim of this study was to use observational data to investigate if homeopathic product users consumed fewer antibiotics and to describe the characteristics of pre-school children given homeopathic products. Use of homeopathic products was not associated with increased or decreased antibiotic consumption. Up to date, experimental data are needed before homeopathic medicines could be considered as an alternative to antibiotics. The characteristics of mothers giving homeopathic products to their children are similar to those associated with adult self-administration. Further research could investigate the source of homeopathic prescription and reasons for use related to antibiotic use.

## Competing interests

The author(s) declare that they have no competing interests.

## Authors' contributions

LW sought the grant, co-ordinated the study and wrote the drafts of the paper. LW, AH & KN designed the study. JB, JH & ET cleaned and coded the data. KN carried out the statistical analyses. JB & ET provided expert opinion on the homeopathy product data, while AH provided expert opinion on the antibiotic data. AH, KN, JH & ET helped with interpretation of the data. AH & KN critically reviewed drafts of the manuscript. All members of the team met regularly on a six weekly basis to review progress. All authors read and approved the final draft.

## Pre-publication history

The pre-publication history for this paper can be accessed here:


